# Etiology, incidence and clinical manifestations of urinary tract infections in evolving population of kidney transplant recipients – a single center 10-year retrospective analysis

**DOI:** 10.3389/fcimb.2026.1831429

**Published:** 2026-09-30

**Authors:** Justyna E. Gołębiewska, Krystian Stefański, Krzysztof Pastuszak, Alicja Dębska-Ślizień

**Affiliations:** 1 Department of Nephrology, Transplantology and Internal Medicine, Medical University of Gdańsk, Gdańsk, Poland; 2 Students’ Scientific Circle, Medical University of Gdańsk, Gdańsk, Poland; 3 Department of Algorithms and System Modelling, Gdańsk University of Technology, Gdańsk, Poland; 4 Department of Translational Oncology, Medical University of Gdańsk, Poland, Gdańsk, Poland; 5 Center of Biostatistics and Bioinformatics, Medical University of Gdańsk, Gdańsk, Poland

**Keywords:** antimicrobial resistance, immunosuppression, kidney transplant, risk factors, urinary tract infections

## Abstract

**Background:**

The aim of the study was to verify how the kidney transplant (KTx) recipient profile changed between 2013 and 2022 and whether these changes influence the incidence, clinical manifestations, and microbiology of urinary tract infections (UTIs).

**Material and methods:**

We analyzed clinical data and urine cultures collected during the first posttransplant year in patients who underwent KTx between January 2013 and December 2022.

**Results:**

We studied data from 491 patients, who developed 448 episodes of UTIs, including asymptomatic bacteriuria (n=211, 47%), lower UTIs (n=80, 18%), acute graft pyelonephritis (n=97, 22%) and urosepsis (n=60, 13%). During two 5-year time periods (2013–2017 and 2018-2022), the number of recipients aged 70 years or over and 75 years or over increased (p=0.07). Recipients receiving a transplant more recently had higher numbers of mismatching HLA (p=0.001) and maximum CDC-PRA (p=0.001). As a result, in 2018–2022 KTx recipients were more likely to receive induction therapy vs. no induction and tacrolimus vs. cyclosporine-based maintenance immunosuppression (p<0.001). There was a parallel change in the etiology of UTIs, from *Escherichia coli* being the predominant pathogen (responsible for almost 35% of all UTI episodes in 2013-2017) to *Klebsiella pneumoniae*, which was isolated in 45% of all UTI episodes between 2018-2022. *Klebsiella pneumoniae* increased over calendar time (OR/year 1.23, 95% CI 1.08–1.39; p=0.0018). The proportion of highly resistant strains rose from 40.1% in 2013-2017, to 53.4% between 2018-2022 (p=0.008) and also increased in the linear model (p = 0.0369).

**Conclusion:**

Over the 10-year period, there was a noticeable change in both KTx recipient and uropathogen characteristics, which requires alteration of antimicrobial prophylaxis and treatment strategies.

## 1 Introduction

The societal aging resulting from increased longevity is accompanied by both increased morbidity and the expectation to maintain good health and high quality of life (Donner et al., 2016). Kidney transplantation (KTx) is the renal replacement therapy of choice for most patients with end-stage kidney disease, providing survival and quality of life benefits in comparison to dialysis. Advances in transplantation success together with the desire of the aging population to live in good health for as long as possible has resulted in a change in KTx recipient characteristics over the last years. This has included an increased percentages of recipients aged 65 years or older, recipients with multiple comorbidities, high immunological risk recipients or recipients of kidney retransplants (Boenink et al., 2023). This trend has been similar in many countries (Park et al., 2020; Ng et al., 2024). It has been shown that elderly patients and retransplant recipients still experience favorable outcomes (Boenink et al., 2023; Ng et al., 2024; Redfield et al., 2015). On the other hand, with improving survival on dialysis, KTx may be regarded as a riskier treatment option in such patients, due to complications and post-transplant morbidity (Pravisani et al., 2021). Urinary tract infections (UTIs) are the most common complications in KTx recipients (Van Delden et al., 2020; Jackson et al., 2021) and account for substantial emergency department use and hospital readmissions in this population (Lovasik et al., 2018). According to the latest United States Renal Data System (USRDS) report, between 2012 and 2021, the rate of infection-related hospital stays in the first year after KTx was three times higher than the rate of cardiovascular disease-related hospital admissions, and among adult KTx recipients, the rates for both types of hospitalizations increased with age (Johansen et al., 2025).

Antimicrobial resistance has become and increasing public health threat, especially to vulnerable populations such as older adults (Murray et al., 2022; Naghavi et al., 2024; Theodorakis et al., 2024), patients with multiple comorbidities (Su et al., 2018; Pailhoriès et al., 2022), and immunocompromised patients, including solid organ transplant recipients (Falcone et al., 2022). These are patients at the highest risk of exposure to both antibiotic treatment and various pathogens due to hospital admissions, often prolonged hospital stay, or the need for multiple invasive interventions. While the prevalence of opportunistic infections in solid organ transplant recipients is decreasing, infectious complications are on the rise, with a visible shift toward multi-drug resistant causative agents, especially *Enterobacterales* (Serris et al., 2023; Bodro et al., 2013; Oriol et al., 2017; Van Delden et al., 2020). Therefore, the aim of the present study was to determine how the patient profile has changed and whether these changes have influenced the incidence, clinical manifestations, microbiology of UTIs including the prevalence of highly resistant strains.

## 2 Materials and methods

We conducted a retrospective cohort study reviewing the medical records of patients who received a kidney allograft transplant at Gdańsk Transplantation Centre, a Polish university affiliated adult KTx center, from the 1^st^ of January 2013 to the 31^st^ of December 2022. According to current kidney allocation policies in Poland, there is one national kidney transplant waiting list, while kidneys are transplanted by the same center that made the procurement. One month after KTx,recipients may transfer to a transplant center of their choice for follow-up (The choice of a center is based on its location, accessibility and travel time). Patients who transferred to another center, returned to dialysis, or died within 1 year after KTx due to reasons other than UTI, were excluded from the analysis.

The following patient-related variables were collected and analyzed:

• age at KTx,
• sex,
• etiology of end-stage kidney disease,
• BMI,
• comorbidity (estimated with the use of the Charlson Comorbidity Index (CCI)) (Charlson et al., 1987),
• pretransplant dialysis time and dialysis modality,
• retransplantation,
• delayed graft function (DGF),
• acute rejection (AR),
• calcineurin inhibitor used (cyclosporine vs. tacrolimus),
• the use of induction therapy with monoclonal (basiliximab) and polyclonal antibodies (antithymocyte globulin),
• CMV infections,
• CDC-PRA last and maximum,
• number of mismatching HLA.

To determine how the patient profile in our population has changed, we compared patient characteristics between two 5-year periods: 2013–2017 and 2018–2022. All variables were also analyzed continuously in relation to the transplant year across the full 2013–2022 study period.

The study was approved by the Medical University of Gdańsk Bioethics Committee (KB/742/2023-2024).

### 2.1 Immunosuppression

All patients initially received a triple maintenance immunosuppressive regimen that consisted of calcineurin inhibitor (tacrolimus (tac) or cyclosporine (CsA)), mycophenolate mofetil (MMF)/sodium (MPS) + glucocorticosteroids. Selected KTx recipients received monoclonal (basiliximab) or polyclonal antibodies (antithymocyte globulin). The choice of the induction agent versus no induction was based on the assessed risk of rejection. Patients with low immunological risk (no PRA, high HLA match, first transplant, and short cold ischemia time) received no induction agent, while patients at high immunological risk (high PRA, high HLA mismatch, retransplant, and long cold ischemia time) receive lymphocyte-depleting agents. If the risk was stratified as intermediate, basiliximab was used.

### 2.2 Prophylaxis for infections

All patients received ceftriaxone perioperatively for 4 days. This antibiotic was selected by the hospital’s epidemiologist, as it takes account of current antibiotic resistance of Gram negative strains. In the case of positive donor’s cultures (urine and/or blood and/or tracheal aspirate), an antibiotic was chosen according to susceptibility profiles. A double-J ureteral stent and a urethral catheter were inserted routinely in all patients. These were removed after 4–5 weeks and between the fourth and fifth day posttransplantation, respectively. Either asymptomatic bacteriuria or a symptomatic UTI was considered an indication for premature double-J ureteral stent removal. To prevent *Pneumocystis jiroveci* infection all patients received 80/400mg of trimetoprim/sulfamethoxazole daily for 6 months. No other urinary prophylaxis was used.

### 2.3 Post-transplant UTI follow-up

Urine cultures were systematically conducted every week within the first month after KTx. Afterward, at each outpatient visit, patients were asked about the presence of UTI symptoms, and basic laboratory tests were obtained including urine analysis, serum creatinine and eGFR. If clinical symptoms suggested UTI or for patients awaiting kidney allograft biopsy or a urological intervention with an anticipated breach of the mucosa, a urine culture was performed. In patients with a history of a UTI with a carbapenemase-producing strain, follow-up urine culture were taken at the discretion of a treating physician to confirm persistent colonization.

### 2.4 Definitions of UTIs

All UTIs were classified into one of four categories: (1) asymptomatic bacteriuria (AB), (2) lower UTI, (3) acute graft pyelonephritis (AGPN), (4) urosepsis. AB was defined according to the guidelines of the Infectious Diseases Society of America (IDSA) as isolation of a bacterial strain in quantitative counts ≥ 10^5^ colony-forming units (CFU) in a clean-catch voided urine specimen in the absence of any symptoms of lower or upper UTI (in women, in two consecutive specimens, the second obtained after at least 24 hours) or < 10^5^ CFU in patients treated with antibiotics or ≥ 10^2^ CFU in a single catheterized urine specimen (Nicolle et al., 2019). Lower UTI was defined as bacteriuria accompanied by dysuria, frequency or urinary urgency and fever < 38°C in the absence of AGPN criteria. AGPN was characterized by significant bacteriuria, fever > 38° and/or graft pain and/or acute kidney allograft injury (Goldman and Julian, 2019). Urosepsis was diagnosed with simultaneous positive blood and urine cultures with the isolation of the same bacterial strain.

Cases of symptomatic UTIs were considered cured with the complete resolution of clinical and a return of lab results to the patient’s preinfection baseline. Patients with AB received no antibiotic treatment unless before a urological procedure with a breach of urinary mucosa.

### 2.5 Pathogen identification

Pathogen identification was determined using a VITEK^®^ 2 Compact (bioMérieux, Marcy l’Étoile, France) until 2020, when it was replaced with a matrix-assisted laser desorption/ionization time of flight (MALDI-TOF) system (Bruker Daltonik, GmbH, Bremen, Germany). Antimicrobial susceptibility testing was performed using the disk diffusion test and interpreted according to current European Committee on Antimicrobial Susceptibility Testing (EUCAST) recommendations. ESBL production was confirmed using E-test (Etest^®^ strips, bioMérieux, Marcy l’Étoile, France) and carbapenemase production was confirmed with a *b*-CARBA^®^ test (Bio-Rad Laboratories, Marnes-la-Coquette, France).

### 2.6 Statistical analysis

Descriptive analyses were performed using Statistica 13 (StatSoft, Poland); regression and calendar-year analyses were performed in R version 4.5.3. The number of nonmissing values, means, standard deviations (SD), ranges, and medians were calculated for numerical variables. The number and percentage were calculated for categorical variables. Continuous variables were compared using Student’s unpaired t test or the Mann-Whitney U test and categorical variables with χ^2^ test or Fisher’s exact test, when appropriate. Transplant year was modeled continuously. A linear year term estimated monotonic annual change, while a natural cubic spline with 3 degrees of freedom modeled the calendar-year association. For each spline fit, a 3-df robust Wald test assessed the overall association and a 2-df robust Wald restriction test assessed nonlinearity versus the nested linear model. Binary transplant-record outcomes used logistic regression, symptomatic-episode rates used negative-binomial regression, continuous characteristics used linear regression, and episode-level outcomes used generalized estimating equations with robust standard errors clustered by transplant record. FDR correction was applied within the predefined families of overall spline tests; nonlinearity p values were not multiplicity-adjusted. Model-predicted annual probabilities or rates with 95% CIs were calculated for the principal outcomes.

Logistic regression analyses were fitted to find independent risk factors for UTIs. A fixed multivariable model included sex, urinary-flow impairment, duration of the initial transplant hospital stay, and Charlson Comorbidity Index (CCI); all variables were entered simultaneously and retained irrespective of p value. The same formula was used in logistic regression for at least one first-year symptomatic UTI and in negative-binomial regression for the total number of recorded symptomatic UTI episodes per transplant record, with record-specific observed follow-up as an offset. Model stability was assessed using 1000 record-level bootstrap samples and leave-one-cohort-year-out analyses. Associations are given as odds ratios (ORs) with a 95% confidence interval (95% CI). Incidence rate ratios (IRRs) with 95% CIs are reported for the count model. Kaplan-Meier curves were plotted to estimate UTI-free survival stratified by variables identified as risk factors in the univariate logistic regression analysis. Statistical tests were two-sided with p values <0.05 considered significant.

## 3 Results

### 3.1 Population and epidemiology of urinary tract infections

From January 1st 2013 to December 31st 2022, 1075 kidney allograft transplantations were performed at Gdańsk Transplantation Center. Data from 491 KTx recipients were included in the analysis (**Figure 1**). Overall, 187 of the remaining 491 patients developed 448 episodes of UTIs including 211 (47%) episodes of AB, 80 (18%) episodes of lower UTI, 97 (22%) episodes of AGPN and 60 (13%) episodes of urosepsis. In 66 patients only episodes of AB were recorded, while 121 patients developed various types of symptomatic infections, including 57 patients who had recurrent symptomatic infections. AB accounted for over 69% of UTI during first posttransplant month, while this proportion decreased to 40% starting from the second posttransplant month.

**Figure 1:**
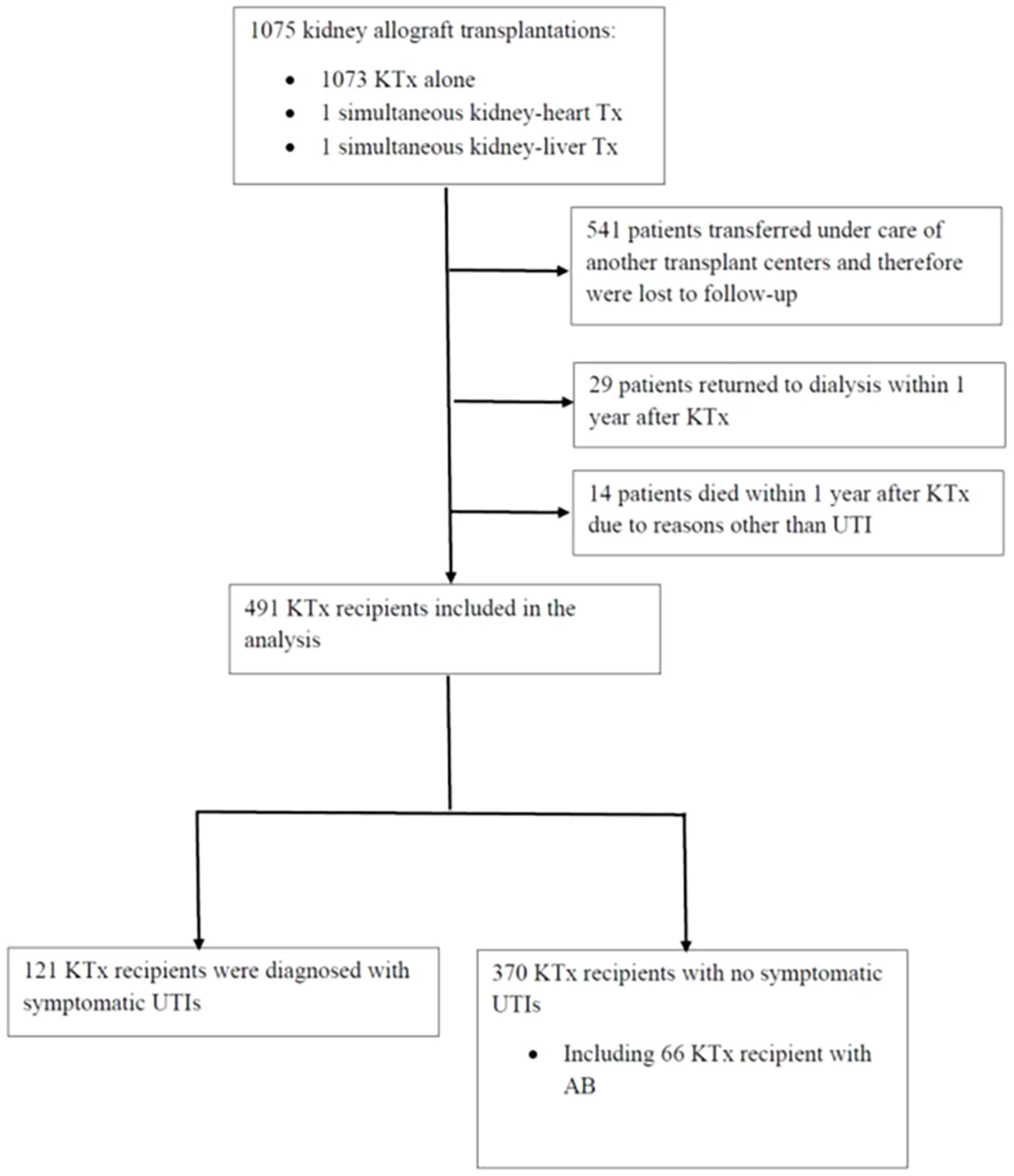
Patient flow diagram.

The baseline characteristics of patients with and without symptomatic UTIs are shown in **Table 1**.

**Table 1:** Demographic and clinical features of patients with and without symptomatic UTIs.

Variable, n (%)	Symptomatic UTI + (n=121)	Symptomatic UTI - (n=370)	p
Age (years)	50.02 ± 14.53	47.80 ± 13.81	0.11
Sex (F/M)	53/68 (44%/56%)	116/254 (31%/69%)	0.02
Etiology of CKD			
Diabetic kidney disease	12 (9.9%)	41 (11.1%)	0.87
Glomerulonephritis	28 (23.1%)	135 (36.5%)	0.008
ADPKD	22 (18.2%)	43 (11.6%)	0.09
Vasculitis	2 (1.7%)	14 (3.8%)	0.38
Congenital urinary abnormalities other than ADPKD	9 (7.4%)	15 (4.1%)	0.15
Unknown etiology	27 (22.3%)	58 (15.7%)	0.1

Comorbidity (CCI) (points)	4.01 ± 1.73	3.59 ± 1.62	0.013
Patients with CCI ≥ 4 points	67 (55.4%)	162 (43.8%)	0.028
BMI (kg/m^2^)	25.29 ± 4.16	25.33 ± 3.92	0.91
RRT prior to KTx			
HD before KTx	90 (74%)	271 (73%)	0.9
PD before KTx	37 (30.6%)	85 (23.0%)	0.11
Preemptive KTx	9 (7%)	50 (14%)	0.08
Dialysis vintage before KTx (months)	31.33 ± 31.10	24.92 ± 25.64	0.013
Retransplant	25 (20.7%)	53 (14.3%)	0.11

Living donor KTx	6 (5%)	25 (6.8%)	0.66
Number of HLA mismatches	2.88 ± 1.16	2.89 ± 1.05	0.78
Max CDC-PRA	7.62 ± 15.28	6.23 ± 13.68	0.13
Last CDC-PRA	3.39 ± 9.41	3.03 ± 10.92	0.14
CIT (minutes)	884.95 ± 365.88	878.40 ± 395.58	0.7
WIT (minutes)	32.1 ± 10.21	29.93 ± 8.57	0.04
AR	8 (6.6%)	15 (4%)	0.32
DGF	45 (37.2%)	86 (23.2%)	0.004
CMV infection	47 (38.8%)	109 (29.5%)	0.057
Urine flow impairment	43 (35.5%)	44 (11.9%)	<0.001
Maintenance immunosuppression			
CsA	34 (28.1%)	83 (22.4%)	0.22
Tac	87 (71.9%)	283 (76.5%)	0.33
MMF/MPS	119 (98.4%)	367 (99.2%)	0.6
Induction therapy			
ATG	47 (39%)	100 (27%)	0.02
basiliximab	40 (33%)	136 (36.8%)	0.51
Induction (either ATG or basiliximab)	86 (71%)	231 (62%)	0.1

Length of KTx hospital stay (days)	22.40 ± 12.08	16.99 ± 7.16	<0.001

ADPKD, autosomal dominant polycystic kidney disease; AR, acute rejection; ATG, antithymocyte globulin; BMI, body mass index; CCI, Charlson Comorbidity Index; CDC-PRA, complement dependent cytotoxicity panel of reactive antibodies; CKD, chronic kidney disease; CIT, cold ischemia time; CMV, cytomegalovirus; CsA, cyclosporine; DGF, delayed graft function; HD, hemodialysis; KTx, kidney transplantation; MMF/MPS, mycophenolate mofetil/sodium; PD, peritoneal dialysis; RRT, renal replacement therapy; tac, tacrolimus; WIT, warm ischemia time.

The incidence rates of UTIs and clinical manifestations did not differ significantly between the 5-year periods (2013–2017 and 2018-2022). Out of 217 patients who received KTx between 2013-2017, 89 patients (41%) developed a type of UTIs including AB episodes in comparison to 98 out of 274 (36%) transplanted between 2018 -2022 (p = 0.26). The proportions were similar when only symptomatic UTIs were considered: 54 out of 217 patients (25%) between 2013 and2017 and 67 out of 274 patients (24.5%) between 2018 and2022 developed various types of symptomatic UTIs (p = 0.92). We also did not find any significant differences when we compared the number of UTI episodes by the infection type (AB, lower UTI, upper UTI, urosepsis) between the two periods. For example, 214 episodes of bacteriuria were recorded in 2013–2017 and 234 in 2018-2022, these included 41 (19%) and 56 (24%) episodes of AGPN (p = 0.25) and another 25 (12%) and 35 (15%) episodes of urosepsis (p = 0.33), respectively.

During the 10-year observation period, only one patient died within the first posttransplant year as a result of *Klebsiella pneumoniae* urosepsis. We did not register any donor-derived UTIs.

### 3.2 Risk factors for symptomatic UTIs and comparison of UTI free survival according to identified risk factors

On univariate analysis, factors significantly associated with symptomatic UTIs were female sex, comorbidity measured by CCI, dialysis vintage, longer warm ischemia time, delayed graft function, use of ATG induction, urine-flow impairment, and prolonged initial KTx hospital stay; CMV infection was more frequent among recipients with symptomatic UTIs than among those without (38.8% vs 29.5%), but the difference was not statistically significant (p = 0.057). In the fixed multivariable logistic model, female sex, urine-flow impairment, and longer KTx hospital stay were associated with symptomatic UTI, whereas total CCI was not associated with the outcome (**Table 2**). To account for recurrent infections, the total number of recorded symptomatic UTI episodes per transplant record was analyzed using negative-binomial regression; the adjusted estimates were female sex (aIRR 1.92 (1.28 to 2.88); p = 0.0016), urine-flow impairment (aIRR 4.09 (2.57 to 6.51); p < 0.001), hospital stay per day (aIRR 1.03 (1.01 to 1.05); p < 0.001), total CCI per point (aIRR 1.01 (0.90 to 1.14); p = 0.8110) (**Table 2**). All 1000 bootstrap fits converged; sign agreement was at least 99.5% for the three associations shared by both models, and their directions were unchanged in all leave-one-cohort-year-out analyses. However, 1-year UTI-free survival also differed significantly after stratification by all factors significantly associated with UTIs, even in a univariate analysis. The cumulative incidence of UTI was lower in men, patients with no urine flow impairment, patients without significant comorbidities (CCI ≤ 3 points) or those who did not receive ATG induction (**Figures 2A–D**). Among patients with recurrent UTIs, 52% had a type of urine flow impairment including prostate hypertrophy, vesicoureteral reflux, or strictures at the vesicoureteral junction. While age was not a risk factor for symptomatic UTIs per se, patients with urine flow impairment were significantly older than those with normal urine flow (55.8 ± 12.5 years vs. 46.8 ± 13.8 years, p <0.001). Longer length of KTx hospital stay possibly reflected a surgical complication increasing the risk of UTI. No significant risk factors were identified among immunosuppressive agents, apart from the use of lymphocyte-depleting induction in univariate analysis. Repeat KTx or HLA sensitization reflected by high CDC-PRA counts did not emerge as risk factors.

**Table 2:** Univariable and multivariable analyses of risk factors associated with at least one first-year symptomatic UTI and recorded symptomatic-episode burden.

Variable	Univariable OR (95% CI; p)	Multivariable aOR (95% CI; p)	Multivariable NB aIRR (95% CI; p)
Age (years)	1.01 (1.00 to 1.03); p = 0.13	–	–
Sex (F/M)	1.71 (1.13 to 2.61); p = 0.01	2.38 (1.48 to 3.81); p < 0.001	1.92 (1.28 to 2.88); p = 0.0016
Comorbidity (CCI) (points)	1.16 (1.03 to 1.31); p = 0.02	1.05 (0.92 to 1.20); p = 0.4791	1.01 (0.90 to 1.14); p = 0.8110
BMI (kg/m2)	1.00 (0.95 to 1.05); p = 0.91	–	–
RRT prior to KTx			
HD before KTx	1.06 (0.66 to 1.69); p = 0.81	–	–
PD before KTx	1.48 (0.93 to 2.33); p = 0.1	–	–
Preemptive KTx	0.51 (0.24 to 1.28); p = 0.06	–	–
Dialysis vintage before KTx (months)	1.01 (1.001 to 1.02); p = 0.03	–	–
Retransplant	1.56 (0.92 to 2.64); p = 0.11	–	–

Living donor KTx	0.72 (0.29 to 1.8); p = 0.47	–	–
Number of HLA mismatches	1.00 (0.84 to 1.19); p = 0.98	–	–
Max CDC-PRA	1.01 (0.99 to 1.02); p = 0.36	–	–
Last CDC-PRA	1.00 (0.98 to 1.02); p = 0.75	–	–
CIT (hours)	1.00 (0.97 to 1.03); p = 0.87	–	–
WIT (minutes)	1.03 (1.003 to 1.05); p = 0.03	–	–
AR	1.68 (0.69 to 4.06); p = 0.27	–	–
DGF	1.96 (1.26 to 3.04); p = 0.003	–	–
CMV infection	1.52 (0.99 to 2.34); p = 0.06	–	–
Urine flow impairment	4.08 (2.5 to 6.66); p < 0.001	3.91 (2.24 to 6.82); p < 0.001	4.09 (2.57 to 6.51); p < 0.001
Maintenance immunosuppression			
CsA	1.35 (0.85 to 2.16); p = 0.21	–	–
Tac	0.79 (0.49 to 1.25); p = 0.31	–	–
MMF/MPS	0.49 (0.08 to 2.99); p = 0.45	–	–
Induction therapy			
ATG	1.71 (1.11 to 2.64); p = 0.02	–	–
basiliximab	0.85 (0.55 to 1.31); p = 0.46	–	–
Induction (either ATG or basiliximab)	1.48 (0.95 to 2.31); p = 0.08	–	–

Length of KTx hospital stay (days)	1.06 (1.04 to 1.09); p < 0.001	1.05 (1.03 to 1.08); p < 0.001	1.03 (1.01 to 1.05); p < 0.001

OR, odds ratio; aOR, adjusted odds ratio; aIRR, adjusted incidence rate ratio; CI, confidence interval; NB, negative binomial;, indicates a predictor not included in the multivariable model. AR, acute rejection; ATG, antithymocyte globulin; BMI, body mass index; CCI, Charlson Comorbidity Index; CDC-PRA, complement dependent cytotoxicity panel of reactive antibodies; CKD, chronic kidney disease; CIT, cold ischemia time; CMV, cytomegalovirus; CsA, cyclosporine; DGF, delayed graft function; HD, hemodialysis; KTx, kidney transplantation; MMF/MPS, mycophenolate mofetil/sodium; PD, peritoneal dialysis; RRT, renal replacement therapy; tac, tacrolimus; WIT, warm ischemia time.

**Figure 2:**
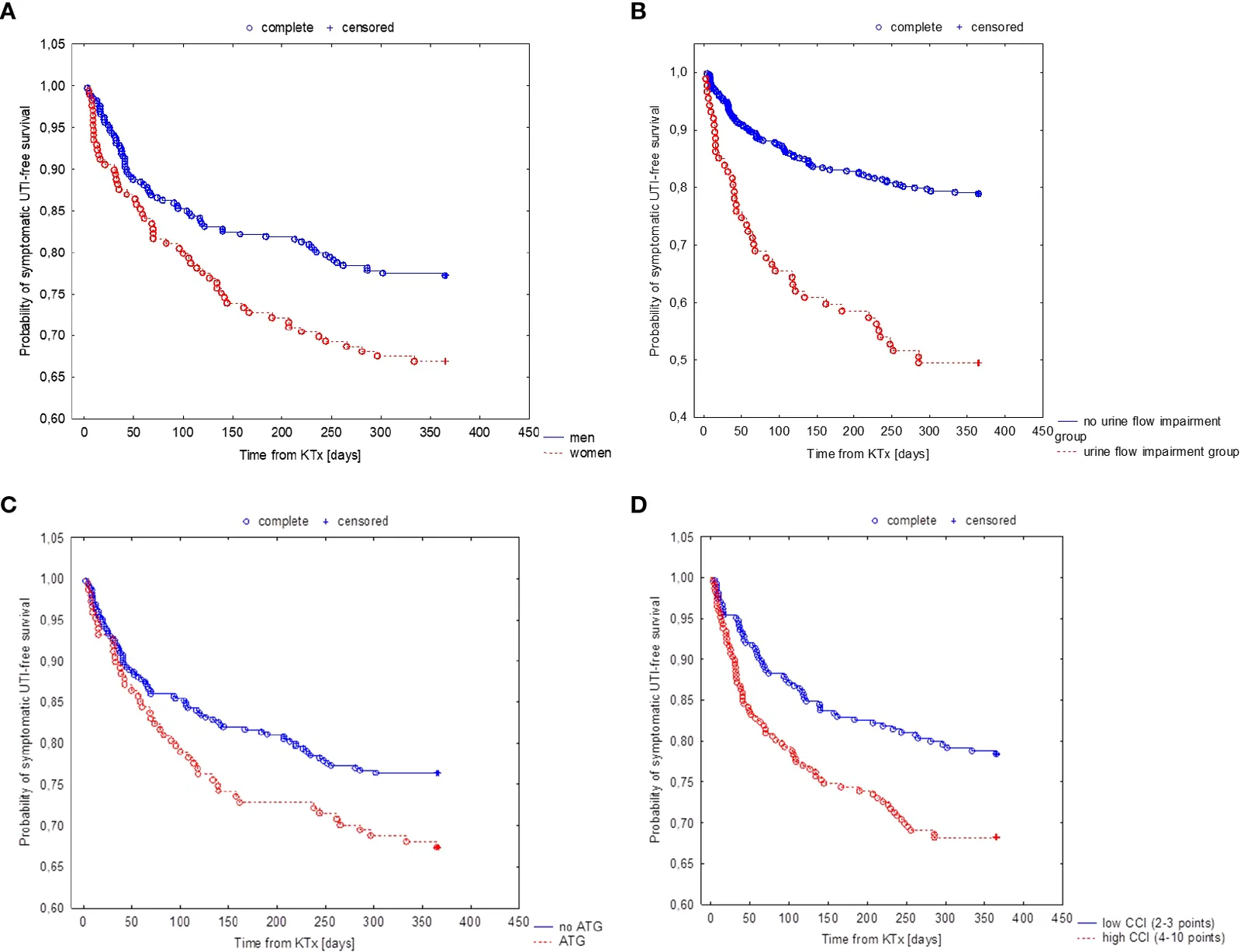
**(A)** Kaplan-Meier survival curve showing symptomatic UTI-free survival in women vs. men (p=0.01). **(B)** Kaplan-Meier survival curve showing symptomatic UTI-free survival in urine flow impairment group vs. no urine flow impairment group vs. no urine flow impairment group (p<0.001). **(C)** Kaplan-Meier survival curve showing sympotomatic UTI-free survival in ATG group vs. no ATG group (p=0.048). **(D)** Kaplan-Meier survival curve showing sympotomatic UTI-free survival in the low Charlson comorbidity index (CCI) (2-3 points) group vs. the high CCI (4-10 points) group (p = 0.006).

### 3.3 Microbiology and antibiotic resistance

The most frequently isolated pathogen was *Klebsiella pneumoniae* (n=153), followed by *Escherichia coli* (n = 121), *Enterococcus faecium* (n = 47) and *Enterococcus faecalis* (n = 43). Causative agents differed by the phenotype of UTI, with *E. coli* being the main pathogen in cases of urosepsis and *Klebsiella pneumoniae* in cases of AB, cystitis and AGPN (**Figure 3**). In 11 UTI episodes two pathogens with comparable numbers were isolated; in most cases one of these were either *E. coli* or *Klebsiella pneumoniae*.

**Figure 3:**
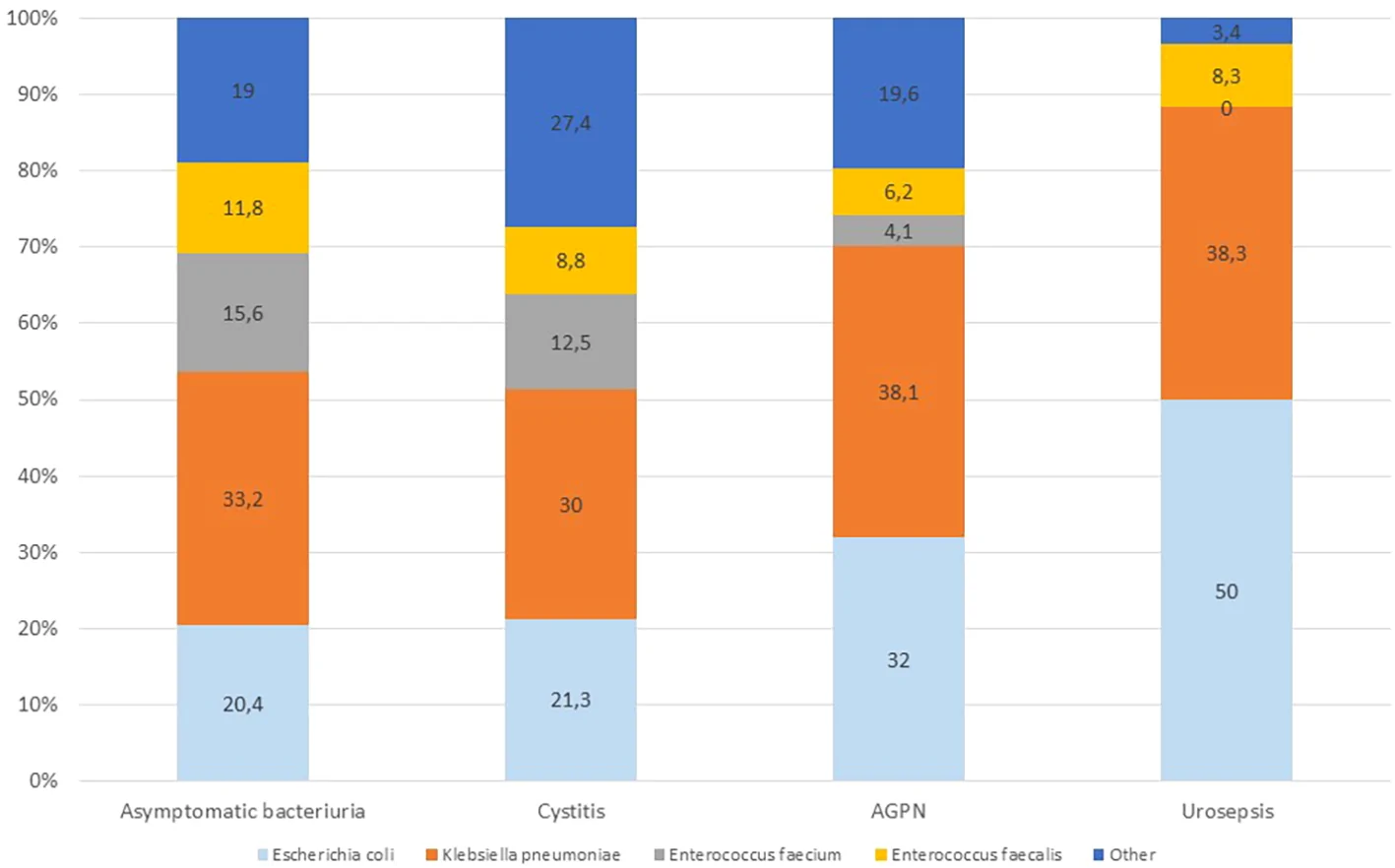
Proportion in different causative agents according to the type of UTI.

We isolated highly resistant strains in 212 cases: ESBL-producing *Enterobacteriaceae* (n = 185), mostly *K. pneumoniae* strains, vancomycin- resistant *E. faecium* (VRE) (n = 13) and *K. pneumoniae* carbapenemase producing strains (n = 14) including 10 MBL-VIM (Verona Integron-encoded metallo-beta-lactamase) strains.

Etiology and antibiotic resistance did not differ significantly between the first posttransplant month and later periods.

Over the whole study period we identified 237 symptomatic UTIs, including 115 (48,5%) caused by highly resistant strains. In slightly over 50% (61/121) of patients with symptomatic UTIs, the causative agent of at least one of the UTI episodes was classified as highly resistant. We did not identify any statistically significant differences in terms of demographic and clinical characteristics when we compared patients who developed symptomatic UTIs with highly resistant strains to those who developed symptomatic UTIs with susceptible strains.

### 3.4 Comparison of patients in 2013–2017 and 2018-2022.

During the two 5-year time periods, 217 and 274 patients, respectively, underwent KTx and remained under our care for at least a year. There were no major differences in the proportion of male vs. female patients, the underlying cause of CKD, dialysis modality before KTx, burden of comorbidity assessed with CCI or number of recipients of a retransplant. While the recipients’ mean age did not differ significantly between the time periods, the number of recipients over 70 or 75 years of age was higher in 2018–2022 than in 2013-2017; however, the difference was not statistically significant. The main difference was the higher immunological risk for more recent patients, as measured by the number of mismatching HLA antigens (p=0.001) and maximum CDC-PRA (p=0.001). As a result, KTx recipients between 2018 and 2022 were more likely to receive both lymphocyte depleting and non-lymphocyte depleting induction therapy versus no induction and tacrolimus-based maintenance immunosuppression vs. cyclosporine-based immunosuppression (p<0.001). Also, warm ischemia time increased, while the length of the initial hospital stay decreased significantly. A detailed comparison is presented in **Table 3**.

**Table 3:** Demographic and clinical features of patients transplanted between 2013–2017 and 2018-2022.

Variable, n (%)	2013-2017 (n=217)	2018-2022 (n=274)	p
Age (years)	47.97 ± 14.14	48.65 ± 13.93	0.717
Patients over 65 years	29 (13%)	42 (15%)	0.61
Patients over 70 years	5 (2%)	16 (6%)	0.07
Patients over 75 years	0 (0%)	5 (2%)	0.07
Sex (F/M)	69/148 (31.8%/68.2%)	100/174 (36.5%/63.5%)	0.29
Etiology of CKD			
Diabetic kidney disease	27 (12.4%)	26 (9.5%)	0.31
Glomerulonephritis	69 (31.8%)	94 (34.3%)	0.56
ADPKD	29 (13.4%)	36 (13.1%)	1.0
Vasculitis	7 (3.2%)	9 (3.3%)	1.0
Congenital urinary abnormalities other than ADPKD	11 (5.1%)	13 (4.7%)	1.0

Unknown etiology	35 (16.1%)	50 (18.2%)	0.55
Comorbidity (CCI) (points)	3.76 ± 1.68	3.64 ± 1.63	0.49
Patients with CCI ≥ 4 points	106 (49%)	123 (45%)	0.41
BMI (kg/m^2^)	25.71 ± 4.03	25.01 ± 3.92	0.08
RRT prior to KTx			
HD before KTx	152 (70.0%)	209 (76.3%)	0.12
PD before KTx	58 (26.7%)	64 (23.4%)	0.4
Preemptive KTx	29 (13.4%)	30 (10.9%)	0.49
Dialysis vintage before KTx (months)	28.35 ± 33.34	25.04 ± 21.05	0.86

Retransplant	32 (14.7%)	46 (16.8%)	0.62
Living donor KTx	13 (6.0%)	18 (6.6%)	0.85
Number of HLA mismatches	2.71 ± 1.02	3.03 ± 1.09	0.001
Max CDC-PRA	5.54 ± 12.60	7.39 ± 15.13	0.001
Last CDC-PRA	2.46 ± 8.76	3.64 ± 11.77	0.2
CIT (minutes)	840.48 ± 358.31	912.07 ± 408.43	0.28
WIT (minutes)	27.56 ± 7.71	32.82 ± 9.36	<0.001
AR	12 (5.5%)	11 (4.0%)	0.52
DGF	65 (30%)	66 (24%)	0.15
CMV infection	85 (39.2%)	71 (25.9%)	0.002
Urine flow impairment	42 (19.4%)	45 (16.4%)	0.41
Maintenance immunosuppression			
CsA	82 (37.8%)	35 (12.8%)	<0.001
Tac	131 (60.4%)	239 (87.2%)	<0.001
MMF/MPS	212 (97.7%)	274 (100.0%)	0.02
Induction therapy			
ATG	40 (18.4%)	107 (39.1%)	<0.001
Basiliximab	64 (29.5%)	112 (40.9%)	0.01
Induction (either ATG or basiliximab)	104 (47.9%)	213 (77.7%)	<0.001

Length of KTx hospital stay (days)	20.13 ± 10.39	16.89 ± 7.29	<0.001

ADPKD, autosomal dominant polycystic kidney disease; AR, acute rejection; ATG, antithymocyte globulin; BMI, body mass index; CCI, Charlson Comorbidity Index; CDC-PRA, complement dependent cytotoxicity panel of reactive antibodies; CKD, chronic kidney disease; CIT, cold ischemia time; CMV, cytomegalovirus; CsA, cyclosporine; DGF, delayed graft function; HD, hemodialysis; KTx, kidney transplantation; MMF/MPS, mycophenolate mofetil/sodium; PD, peritoneal dialysis; RRT, renal replacement therapy; tac, tacrolimus; WIT, warm ischemia time.

### 3.5 Changes across transplant year

**Table 4** reports linear annual effects, overall spline tests, and tests of nonlinearity for recipient and transplant characteristics across continuous transplant year. FDR-adjusted overall spline tests supported calendar-year associations for HLA mismatches, maximum and last CDC-PRA, warm ischemia time, tacrolimus use, ATG-containing induction, and initial transplant hospital stay. For last CDC-PRA, there was evidence of an overall calendar-year association (overall spline p = 0.0025; FDR-adjusted p = 0.0102) but not of a monotonic annual change (Mean change/year 0.28 (-0.01 to 0.57); p = 0.0574); the nonlinearity test was null (p = 0.3140). Age thresholds are summarized descriptively and were not tested formally.

**Table 4:** Recipient and transplant characteristics across transplant year.

Characteristic	Overall	Annual effect (95% CI; p)	Overall spline p; FDR p	Nonlinearity p
Age, years	48.35 ± 14.01 (N = 491)	Mean change/year 0.14 (-0.30 to 0.57); p = 0.5425	0.9244; 0.9776	0.9266
Age 65 years or older	71/491 (14.46%)	Descriptive only	Not tested	Not tested
Age 70 years or older	21/491 (4.28%)	Descriptive only	Not tested	Not tested
Age 75 years or older	5/491 (1.02%)	Descriptive only	Not tested	Not tested
Sex, female/male	169/322 (34.42%/65.58%; N = 491)	OR/year 1.08 (1.01 to 1.16); p = 0.0249	0.0310; 0.0899	0.1361
Etiology of chronic kidney disease				
Diabetic kidney disease	53/491 (10.79%)	OR/year 0.93 (0.83 to 1.03); p = 0.1734	0.4253; 0.6297	0.7402
Glomerulonephritis	163/491 (33.20%)	OR/year 1.04 (0.97 to 1.11); p = 0.2777	0.4836; 0.6297	0.5536
ADPKD	65/491 (13.24%)	OR/year 0.98 (0.89 to 1.07); p = 0.6476	0.9507; 0.9776	0.9252
Vasculitis	16/491 (3.26%)	OR/year 1.01 (0.84 to 1.22); p = 0.8975	0.9776; 0.9776	0.9136
Congenital urinary abnormalities other than ADPKD	24/491 (4.89%)	OR/year 0.94 (0.82 to 1.09); p = 0.4446	0.4168; 0.6297	0.3674
Unknown etiology	85/491 (17.31%)	OR/year 1.01 (0.93 to 1.10); p = 0.8252	0.4994; 0.6297	0.3153
Charlson Comorbidity Index, points	3.69 ± 1.66 (N = 491)	Mean change/year -0.02 (-0.08 to 0.03); p = 0.4310	0.5707; 0.6896	0.5442
Charlson Comorbidity Index, 4 points or higher	229/491 (46.64%)	Descriptive only	Not tested	Not tested
Body mass index, kg/m²	25.32 ± 3.98 (N = 491)	Mean change/year -0.11 (-0.24 to 0.01); p = 0.0636	0.0812; 0.2098	0.3211
Renal replacement therapy before transplantation				
Hemodialysis	361/491 (73.52%)	OR/year 1.05 (0.97 to 1.13); p = 0.2077	0.4961; 0.6297	0.7116
Peritoneal dialysis	122/491 (24.85%)	OR/year 0.96 (0.89 to 1.03); p = 0.2200	0.3305; 0.5638	0.3282
Preemptive transplantation	59/491 (12.02%)	OR/year 0.99 (0.89 to 1.10); p = 0.8421	0.4632; 0.6297	0.2963
Dialysis vintage before transplantation, months	26.50 ± 27.20 (N = 491)	Ratio/year 0.99 (0.95 to 1.03); p = 0.7004	0.6844; 0.7634	0.4948
Retransplantation	78/491 (15.89%)	OR/year 0.99 (0.92 to 1.08); p = 0.8542	0.1139; 0.2359	0.0510

Living donor transplantation	31/491 (6.31%)	OR/year 0.98 (0.85 to 1.12); p = 0.7475	0.6827; 0.7634	0.5296
HLA mismatches	2.89 ± 1.07 (N = 491)	Mean change/year 0.06 (0.03 to 0.09); p < 0.001	<0.001; 0.0045	0.1021
Maximum CDC-PRA	6.58 ± 14.08 (N = 490)	Mean change/year 0.61 (0.17 to 1.04); p = 0.0064	<0.001; <0.001	0.1456
Last CDC-PRA	3.12 ± 10.56 (N = 490)	Mean change/year 0.28 (-0.01 to 0.57); p = 0.0574	0.0025; 0.0102	0.3140
Cold ischemia time, minutes	880.03 ± 388.04 (N = 467)	Mean change/year 17.60 (4.08 to 31.13); p = 0.0108	0.0872; 0.2098	0.7324
Warm ischemia time, minutes	30.47 ± 9.04 (N = 481)	Mean change/year 0.97 (0.70 to 1.24); p < 0.001	<0.001; <0.001	0.4451
Acute rejection	23/491 (4.68%)	OR/year 0.93 (0.81 to 1.06); p = 0.2809	0.2861; 0.5185	0.2350
Delayed graft function	131/491 (26.68%)	OR/year 0.95 (0.88 to 1.02); p = 0.1473	0.0940; 0.2098	0.1200
CMV infection	156/491 (31.77%)	OR/year 0.92 (0.86 to 0.99); p = 0.0258	0.0202; 0.0732	0.0871
Urinary-flow impairment	87/491 (17.72%)	OR/year 0.95 (0.87 to 1.04); p = 0.2549	0.1433; 0.2771	0.1622
Maintenance immunosuppression				
Cyclosporine	117/491 (23.83%)	Descriptive only	Not tested	Not tested
Tacrolimus	370/491 (75.36%)	OR/year 1.28 (1.19 to 1.39); p < 0.001	<0.001; <0.001	<0.001
MMF or MPS	486/491 (98.98%)	Descriptive only	Not tested	Not tested
Induction therapy				
ATG	147/491 (29.94%)	OR/year 1.31 (1.21 to 1.42); p < 0.001	<0.001; <0.001	0.0075
Basiliximab	176/491 (35.85%)	OR/year 1.09 (1.02 to 1.17); p = 0.0107	0.0263; 0.0848	0.1895
Either ATG or basiliximab	317/491 (64.56%)	Descriptive only	Not tested	Not tested

Initial transplant hospital stay, days	18.32 ± 8.93 (N = 491)	Ratio/year 0.97 (0.96 to 0.98); p < 0.001	<0.001; <0.001	0.0863

Annual effects are from year-only models; spline columns report overall, FDR-adjusted, and nonlinearity p values. Effects are OR/year, mean change/year, or ratio/year; age thresholds and overlapping treatment categories are descriptive. ADPKD, autosomal dominant polycystic kidney disease; AR, acute rejection; ATG, antithymocyte globulin; BMI, body mass index; CCI, Charlson Comorbidity Index; CDC-PRA, complement dependent cytotoxicity panel of reactive antibodies; CKD, chronic kidney disease; CIT, cold ischemia time; CMV, cytomegalovirus; CsA, cyclosporine; DGF, delayed graft function; HD, hemodialysis; KTx, kidney transplantation; MMF/MPS, mycophenolate mofetil/sodium; PD, peritoneal dialysis; RRT, renal replacement therapy; tac, tacrolimus; WIT, warm ischemia time.

### 3.6 Comparison of uropathogens in 2013–2017 and 2018-2022

There was a parallel change in the etiology of UTIs: *E. coli* was the predominant pathogen responsible for almost 35% of all UTI episodes between 2013 and 2017, but *K. pneumoniae* isolated in 45% of all UTI episodes between 2018 and 2022 (**Figure 4**). A similar change was visible across all types of UTIs. When we analyzed all UTI episodes, the proportion of episodes caused by *Klebsiella pneumoniae* increased linearly over calendar time (OR/year 1.23, 95% CI 1.08–1.39; p = 0.0018); the overall spline association was also significant after FDR adjustment (overall spline p = 0.0019; FDR-adjusted p = 0.0115), with no evidence of nonlinearity (p = 0.1085). Among symptomatic episodes, the proportion of episodes caused by *K. pneumoniae* increased linearly over calendar time (OR/year 1.28, 95% CI 1.12–1.46; p < 0.001). *E. coli* showed a nonlinear calendar-year association (overall spline p = 0.0049; FDR-adjusted p = 0.0098; p for nonlinearity = 0.0143), while *E. faecium* decreased over calendar time (OR/year 0.75, 95% CI 0.60–0.93; p = 0.0080). The shift in bacterial species isolated from urine cultures was accompanied by a raise in antibiotic resistance. Between 2013 and 2017, 40.1% of UTIs were caused by highly resistant strains, while between 2018 and 2022 this proportion rose to 53.4% (p=0.008). The proportion of symptomatic infections due to highly resistant strains was also higher between 2018 and 2022 at 54.5% (67 out of 123 episodes) versus 42% (48 out of 114 episodes) in the earlier period. However, this change did not reach statistical significance (p = 0.07). When we performed time trend analysis of all UTI episodes highly resistant phenotype increased in the linear model (OR/year 1.12, 95% CI 1.01–1.25; p = 0.0369), with no evidence of nonlinearity (p = 0.7524). Among symptomatic UTI episodes, the proportion of all highly resistant showed an upward trend, but it was not statistically significant (OR/year 1.11, 95% CI 0.98–1.26; p = 0.0958). The number of ESBL-producing strains isolated/year increased over the 10-year observation period among symptomatic UIT episodes in the linear model (OR/year 1.13, 95% CI 1.01–1.26; p = 0.0353) (**Table 5**).

**Figure 4:**
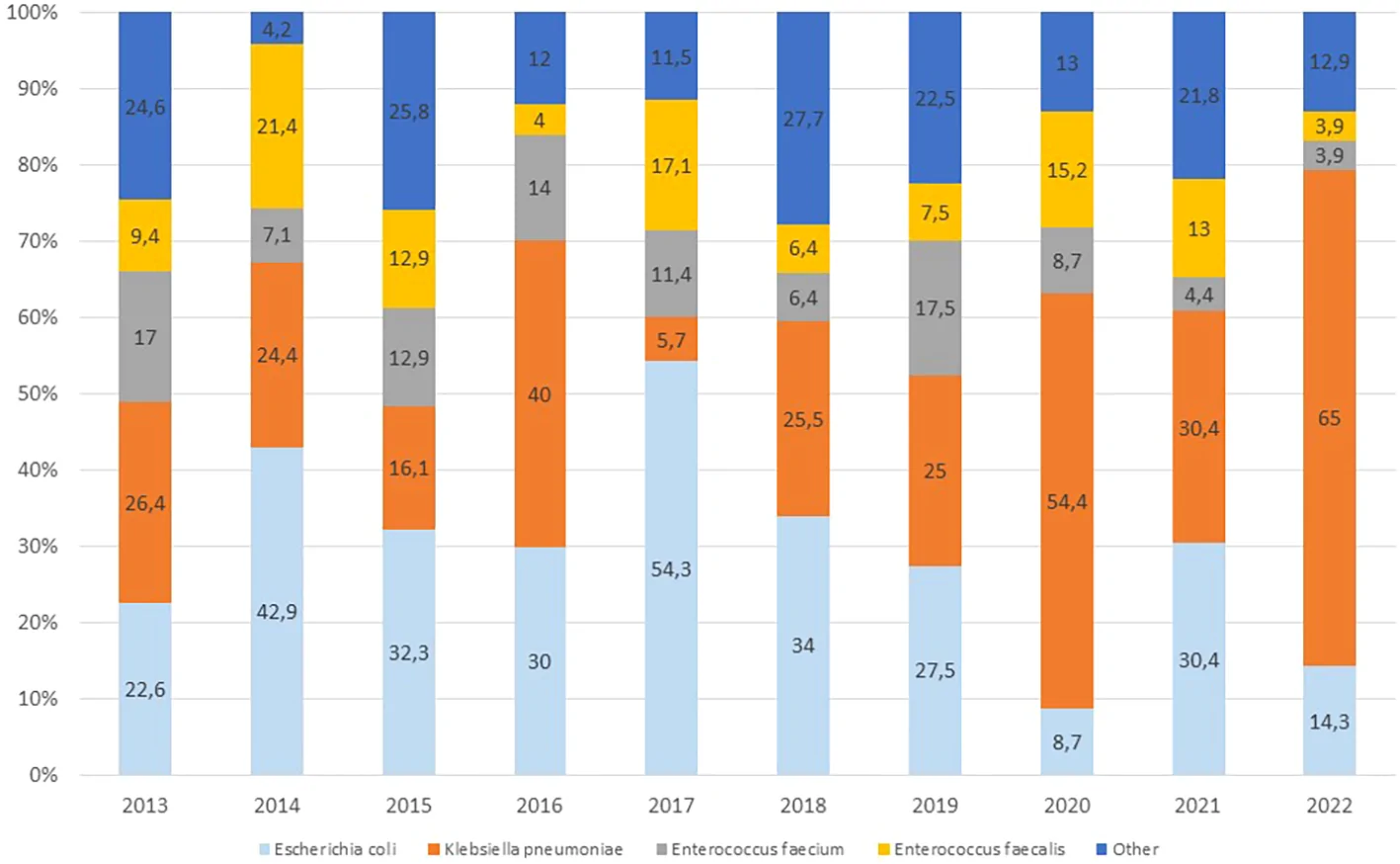
Proportion of different causative agents in all UTIs in years 2013-2022.

**Table 5:** Calendar-year associations for different UTI causative agents.

Outcome	n/N	Annual effect (95% CI; p)	Overall spline p; FDR p	Nonlinearity p
All UTIs				
All highly resistant strains	212/448	OR/year 1.12 (1.01 to 1.25); p = 0.0369	0.1487; 0.2231	0.7524
ESBL-producing strains	185/448	OR/year 1.07 (0.96 to 1.19); p = 0.2144	0.5979; 0.5979	0.8954
*Klebsiella pneumoniae*	153/448	OR/year 1.23 (1.08 to 1.39); p = 0.0018	0.0019; 0.0115	0.1085
*Escherichia coli*	121/448	OR/year 0.91 (0.81 to 1.02); p = 0.1067	0.0479; 0.1437	0.1678
*Enterococcus faecium*	47/448	OR/year 0.88 (0.78 to 0.98); p = 0.0244	0.0892; 0.1784	0.6212
*Enterococcus faecalis*	43/448	OR/year 0.94 (0.84 to 1.06); p = 0.3196	0.5047; 0.5979	0.6081
Symptomatic UTIs				
Highly resistant strains	115/237	OR/year 1.11 (0.98 to 1.26); p = 0.0958	0.3502; 0.4203	0.8689
ESBL-producing strains	108/237	OR/year 1.13 (1.01 to 1.26); p = 0.0353	0.1825; 0.2738	0.9554
*Klebsiella pneumoniae*	83/237	OR/year 1.28 (1.12 to 1.46); p < 0.001	<0.001; 0.0028	0.1336
*Escherichia coli*	78/237	OR/year 0.91 (0.79 to 1.03); p = 0.1393	0.0049; 0.0098	0.0143
*Enterococcus faecium*	14/237	OR/year 0.75 (0.60 to 0.93); p = 0.0080	<0.001; 0.0028	0.0854
*Enterococcus faecalis*	18/237	OR/year 0.95 (0.81 to 1.12); p = 0.5347	0.8339; 0.8339	0.7546

Annual effects are from year-only models; spline columns show overall p, FDR-adjusted p, and nonlinearity p for 3-df natural cubic splines. Episode-level models use logistic generalized estimating equations clustered by transplant record.

All but one carbapenemase-producing strains were registered in the year 2022.

## 4 Discussion

The study examined how the KTx recipient profile changed over the 10 years and whether these changes influenced the incidence, clinical manifestations, microbiology of UTIs.

We found differences in all aspects of the kidney transplantation, including recipient characteristics, immunosuppression regimens, warm ischemia time (WIT) and the length of initial KTx hospital stay. More recent recipients had higher immunological risk reflected by a higher number of mismatching HLA antigens and maximum CDC-PRA. These findings were consistent with reports from Europe based on the Collaborative Transplant Registry Database (Boenink et al., 2023), the Australia and New Zealand Dialysis and Transplant (ANZDATA) Registry (Ng et al., 2024) and the Scientific Registry of Transplant Recipients (SRTR) in the United States (Huang et al., 2017). As a result, between 2018 and 2022, our KTx recipients were more likely to receive more potent immunosuppression protocols, including induction agents and tacrolimus-based maintenance immunosuppression. Warm ischemia time increased over the observation period, which most probably reflecting more complex donor and recipient characteristics, including more advanced vascular disease.

There was also a noticeable trend towards higher numbers of recipients over 70 and 75 years of age. In the United States between 2012 and 2022, the absolute number of KTxs increased among individuals aged 45–64 years and 65–74 years, but there was no visible change in the number of kidneys transplanted to patients aged ≥75 years (Johansen et al., 2025). The analysis of data from the ANZDATA Registry showed that proportion of KTx recipients aged 65 years or older almost tripled over a period of the years from 1995 to 2014 (Ng et al., 2024). According to the European Renal Association Registry data, the proportion of recipients aged 65 years or older also increased, but the size of increase in this age group varied across European countries (Boenink et al., 2023). In a study by Lemoine et al. over 80% of 171 French septuagenarians developed infectious complications during the first post-KTx year, including almost 20% who experienced recurrent UTIs. Furthermore, infections accounted for almost 60% of mortality during the first posttransplant year in this population (Lemoine et al., 2019). In another study on a group of 166 elderly Chinese KTx recipients, infections were the leading cause of death at 5 years, being responsible for over 55% of deaths (Lai et al., 2014).

In spite of the unfavorable changes in KTx recipient characteristics we managed to shorten the mean length of hospital stay.

When we studied the incidence of UTIs, AB episodes predominated in the studied population irrespective of the time period analyzed. AB is nowadays considered a form of colonization where bacteria with reduced virulence attenuate the host immune response in order to avoid the destructive full-blown inflammatory reaction (Ambite et al., 2021). For this reason we decided to focus on symptomatic infections, which can have a deleterious effect on both the recipient and on graft survival (Abbott et al., 2004; Naik et al., 2016; Brune et al., 2022). The independent risk factors for symptomatic UTIs were limited to female sex, impaired urine flow and the length of KTx hospital stay. Both female gender and impaired urine flow are well-known risk factors for UTIs in general population as well. The current Guidelines from the American Society of Transplantation Infectious Diseases Community of Practice recommend an assessment of potential anatomic and functional abnormalities in order to identify structural factors which might predispose to recurrent UTIs in kidney transplant recipients (Goldman and Julian, 2019). While age was not a risk factor for symptomatic UTIs per se, patients with urine flow impairment were significantly older than those with normal urine flow; prostate hypertrophy was one of the most common structural abnormalities identified in patients with recurrent upper UTIs. Other conditions resulting in urine flow impairment were not related to age; for example, vesicoureteral reflux and ureteral or urethral stenosis were much less common in our population and were not reflected in statistical analysis. Contrary to our earlier findings (Gołębiewska et al., 2014), comorbidity measured by CCI was not an independent risk factor for either symptomatic UTIs or UTIs with highly resistant strains. One possible explanation for this inconsistency is that CCI was not designed for use with infectious disease outcomes. In addition, CCI has poor discrimination ability in the setting of VRE or MRSA infections (McGregor et al., 2005).

Parallel to changes in recipient characteristics, there was a shift in the etiology of UTIs, from *E. coli* to *K. pneumoniae* being the predominant pathogen. In our earlier studies and in other Polish transplant centers, *K. pneumoniae* was reported as the second- or third-most commonly isolated uropathogen (Gołębiewska et al., 2014; Adamska et al., 2015; Gozdowska et al., 2016; Rostkowska et al., 2020; Krawczyk et al., 2022). However, over time the incidence of *K. pneumoniae* UTIs has visibly increased. The reported frequency with which *K. pneumoniae* is found in the urine cultures of KTx recipients varies depending on the country, region, transplant center, and study period, with a range from 5% (Valera et al., 2006) to 53% (Silva et al., 2013). The short observation period and small populations investigated can, at least in part explain the variation seen in between earlier reports (Abbott et al., 2004; Dantas et al., 2006; Valera et al., 2006; Gołębiewska et al., 2011; Silva et al., 2013; Adamska et al., 2015; Gozdowska et al., 2016). Unfortunately, no up to date published data are available on the epidemiology of UTIs in Polish KTx recipients for comparison with other KTx centers in Poland.

The reason for this trend in not entirely clear. One potential driver could be nosocomial transmission. However, in our study published in 2019, where we studied genetic relatedness of 61 *Klebsiella pneumoniae* strains collected from 54 KTx recipients, we found 45 unrelated, unique genotypes and 7 related (16 isolates from 15 patients) genotypes (Gołębiewska et al., 2019). The alarming trend of elevated antimicrobial resistance levels has been seen worldwide. *K. pneumoniae* is one of the seven leading pathogens responsible for deaths associated with antimicrobial resistance in the WHO European region (Mestrovic et al., 2022). *K. pneumoniae*, in comparison to *E.coli*, has shown much higher nonsusceptibility rates to all classes of antibiotics throughout the last decade (Navon-Venezia et al., 2017). This could be another driver of the observed microbial shift from *E. coli* to *K. pneumoniae*. Antibiotic resistance is largely driven by antibiotic pressure. Kachalov et al., with the use of a transmission model of cephalosporin- and carbapenem-resistant *K. pneumoniae* parameterized with antibiotic consumption and demographic data from 11 European countries. They showed that nosocomial transmission and antibiotic consumption in the hospital had major impact on ESBL and carbapenem-resistance prevalence, while antibiotic consumption in the community had a minor impact (Kachalov et al., 2021). We use ceftriaxone for perioperative prophylaxis. This is also the antibiotic of choice in the empirical treatment of systemic UTIs as per both our internal hospital guidelines and the guidelines of the European Association of Urology in cases of high fluoroquinolone resistance (EAU, 2025).

The antimicrobial resistance reported in our study mirrored trends seen in Poland and other European Union countries only to an extent. While there was a parallel increase in resistance rates to third-generation cephalosporins and fluoroquinolones among *E. coli* and *K. pneumoniae* strains, the rates in our study were much higher, even approaching 90 or 100% for fluoroquinolones throughout the whole observation period. At the same time, resistance rates to carbapenems and aminoglycosides were much lower than those reported by European Centre for Disease Prevention and Control (ECDC), most probably reflecting our local trends in antibiotic use (ECDC, 2017, 2018, 2020, 2023). What is interesting, although the use of carbapenems was relatively common among our patients due to the high number of infections with ESBL-producing strains, we report only a single infection with carbapenem-resistant strain until 2022. In a recently published analysis of antibiotic resistance in uropathogens isolated from German KTx recipients between 2013 and 2022, the proportion of *K. pneumoniae* strains isolated remained stable over the period, while the authors reported an increase in the proportion of ESBL-producing isolates among *K. pneumoniae* (Weber et al., 2025). However, the latter study analyzed urine samples not infection episodes.

The observed microbiological shift and increasing resistance rates to most commonly used antibiotics require reconsideration of our prophylaxis, treatment, and surveillance strategies. There is an urgent need to apply known antimicrobial stewardship principles and broader use of newly developed molecular rapid diagnostic testing tools in order to ensure more rational antimicrobial prescription and more targeted treatment of infections (Ioannou et al., 2022). Another option would be to shorten antibiotic treatment: a recently published meta-analysis showed that shorter perioperative and briefer treatment duration for uncomplicated kidney allograft infection were safe, as they did not increase the number of infections and reduced antibiotic consumption (Paniagua-García et al., 2025).

KTx recipients are perceived as especially prone to acquire highly resistant bacteria, and in fact the number of infections with highly resistant strains is increasing in this patient population (Dumford and Skalweit, 2020; Pagani et al., 2021). Impaired immunity promotes antimicrobial resistance with a variety of mechanisms, including a lack of innate immune clearance, alterations in native microbial flora, and extensive antibiotic exposure (Margolis and Rosch, 2018). On the other hand, in a prospective multicenter cohort study in an intensive care unit (ICU) setting, immunocompromised patients including solid organ transplant recipients had a lower incidence rate of ICU-acquired colonization with multidrug resistant strains than controls, and the incidence rate of ICU-acquired infection with multidrug resistant strains did not differ between groups (Kreitmann et al., 2023). The authors concluded that this favorable outcome most likely resulted from stricter adherence to infection prevention and control precautions when an increased risk of contact transmission was anticipated. Immunosuppression also had no influence on the incidence of ICU-acquired bacterial bloodstream infections (Zebian et al., 2024) or mortality in carbapenem-resistant bacterial bloodstream infections (Li et al., 2024).

The main limitations of this study is retrospective nature and a relatively small sample size, which is a consequence of the national kidney allocation policy resulting in over half of the recipients changing their follow-up center after 1 month from KTx. Multivariable adjustment may have been limited by a relatively small number of events. Generalization of the presented results may also be limited, as the study was restricted to a single transplantation center with specific surgical techniques used (e.g. a double-J stent is always used) and particular strategies for infection prophylaxis and immunosuppressive treatment. Not all potential confounders were evaluated (e.g. the history of recurrent UTIs before KTx, sexual activity, or everyday habits, etc.). While analyzing the etiology of UTIs, we focused on four most common pathogens in the studied population: the Gram-negative bacteria *E. coli, K. pneumoniae*, and the Gram-positive bacteria *E. faecium* and *E. faecalis*. We did not analyze other pathogens, such as *Pseudomonas aeruginosa* or *Proteus mirabilis*, as these were seldom isolated and would not allow for statistical analysis. Also, the transition from VITEK 2 to MALDI-TOF could have had a minor influence on the results of bacterial identification (Febbraro et al., 2016; Kassim et al., 2017). However, we believe that it would not affect the primary outcomes or the general conclusion. What is more, because data on antimicrobial consumption, colonization surveillance, infection prevention measures, or possible healthcare-associated transmission are lacking, it is difficult to determine whether the observed changes in causative agents and resistance primarily reflect antibiotic selection pressure, nosocomial transmission, or broader epidemiological trends. Despite these limitations, we believe that our study is of importance.

To conclude, recent years have seen a noticeable change in KTx recipient characteristics, center strategies including the administration of various immunosuppressive treatment regimens and attempts to shorten the initial posttransplant hospital stay. In parallel, there has been a shift in uropathogens toward multidrug-resistant. It requires further investigation to determine if this is only a temporal association rather than causal effect. Even if changing recipient characteristics does not influence the incidence and clinical manifestations of UTIs, the changing microbiology may affect recipients with a raising number of difficult-to-treat infections. Raising antimicrobial resistance requires continuous surveillance, a change in the existing antimicrobial prophylaxis, stricter transmission-based precautions, antibiotic stewardship and updated treatment strategies in order to improve outcomes in this vulnerable population.

## Data Availability

The data analyzed in this study is subject to the following licenses/restrictions: The datasets used and/or analyzed during the current study are available from the corresponding author on reasonable request. Requests to access these datasets should be directed to jgolebiewska@gumed.edu.pl.
